# The Roles of RNA N6-Methyladenosine in Regulating Stem Cell Fate

**DOI:** 10.3389/fcell.2021.765635

**Published:** 2021-11-05

**Authors:** Runbi Ji, Xu Zhang

**Affiliations:** ^1^The Affiliated People’s Hospital of Jiangsu University, Zhenjiang, China; ^2^Jiangsu Key Laboratory of Medical Science and Laboratory Medicine, School of Medicine, Jiangsu University, Zhenjiang, China

**Keywords:** N6-methyladenosine, post-transcriptional modifications, cancer, stem cell, cell differentiation

## Abstract

RNA N6-methyladenosine (m^6^A) modification has important regulatory roles in determining cell fate. The reversible methylation process of adding and removing m^6^A marks is dynamically regulated by a fine-tuned coordination of many enzymes and binding proteins. Stem cells have self-renewal and pluripotent potential and show broad prospects in regenerative medicine and other fields. Stem cells have also been identified in cancer, which is linked to cancer metastasis, therapy resistance, and recurrence. Herein, we aimed to review the molecular mechanism that controls the reversible balance of m^6^A level in stem cells and the effect of m^6^A modification on the balance between pluripotency and differentiation. Additionally, we also elaborated the association between aberrant m^6^A modification and the maintenance of cancer stem cells in many cancers. Moreover, we discussed about the clinical implications of m^6^A modification in cancer stem cells for cancer diagnosis and therapy.

## Introduction

Although the definition of stem cells has been constantly revised, the key properties of stem cells are self-renewal and multidifferentiation potency. Stem cells have differentiation plasticity in a specific microenvironment to make up tissues and organs, thus, playing important roles in regenerative medicine and tissue engineering.

The coordinated gene expression for stem cell maintenance and differentiation are established by epigenetic changes (Berdasco and Esteller, 2011). Increasing studies have shown that m^6^A modification, the most abundant form of methylation modification in eukaryotic mRNA, can dynamically regulate the expression balance of pluripotency or lineage development factors, which determines the fate of stem cells (self-renewal or differentiation) (Zhao and He, 2015).

Here, we concentrated on the current advances of m^6^A modification in stem cell self-renewal and differentiation, especially the specific mechanism of regulation. We highlighted the potential of m^6^A machinery as novel strategies for stem cell expansion in tissue regeneration and stem cell clearance in cancer.

## Regulatory Factors for m^6^A Modification

**FIGURE 1:**
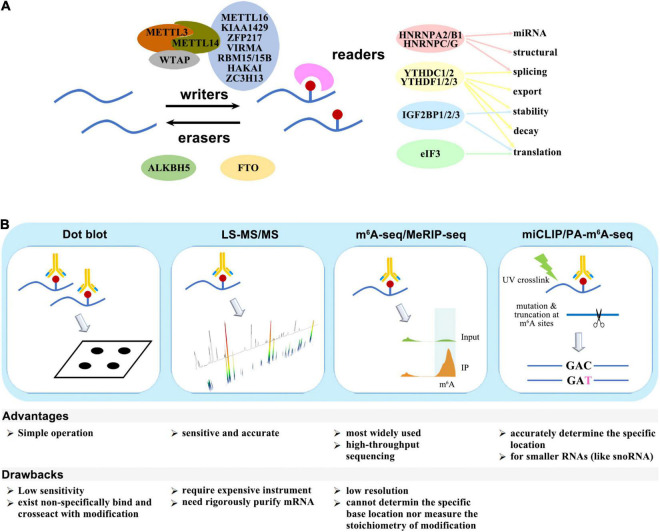
**(A)** Writers, erasers, and readers of the m^6^A modification. **(B)** Schematic diagram of m^6^A detection methods.

The m^6^A modification is deposited by methyltransferase (writers), removed by demethylase (erasers), and recognized by binding proteins (readers). The “writers” include METTL3/5/14/16, KIAA1429, WTAP, VIRMA, HAKAI, RBM15/15B, ZC3H13, and PCIF1/CAPAM, while FTO and ALKBH5 perform as “erasers.” The known “readers” are YTHDF1/2/3, YTHDC1/2, IGF2BP1/2/3, HNRNPA2B1/C/G, eIF3, and FMR1/FMRP. These factors either act independently, or form complexes, or cooperate with other cofactors to participate in almost all steps of RNA metabolism, including structural switch, splicing, export, stability, decay, translation, and miRNA mature (Figure 1A).

## Technology in m^6^A Research

Several quantitative or site-specific methods have been developed to detect the amount or location of m^6^A modification (Figure 1B). The methods for global quantitative measurement of m^6^A include dot blot by using m^6^A-specific antibodies or LC-MS/MS. The procedure for dot blot is relatively simple, but the sensitivity is low, and there is non-specific binding between existing antibodies and other modifications. In comparison, LC-MS/MS is more sensitive and accurate, though it requires expensive instrument.

Methods for detection of the m^6^A modification site include m^6^A−seq, MeRIP-seq, PA-m^6^A−CLIP, miCLIP, m^6^A-LAIC-SEQ, SCARLET, and SELECT. m^6^A−seq and MeRIP-seq is the earliest and most widely used technologies. Purified mRNA fragments are immunoprecipitated by m^6^A-specific antibodies and subjected to high-throughput sequencing, but these methods have a low resolution and cannot measure the stoichiometry. PA-m^6^A−CLIP and miCLIP can accurately determine the specific location of m^6^A modification site. The purified RNA are incorporated with photoactivity ribonucleoside analogs, and the data are analyzed after immunoprecipitation and UV-induced cross-linking.

## m^6^A Modification in Fate Decision of Stem Cells

### Embryonic Stem Cells

Embryonic stem cells (ESCs) are derived from the embryo cell mass or primordial germ cells. They can be induced to differentiate into all kinds of cells in the body and expanded, screened, frozen, and resuscitated *in vitro* without losing their original characteristics. Considerable efforts have been devoted to characterize the epigenetic networks that control the reprogramming of ESCs, which requires precise coordination of transcription factors, chromatin regulators, and RNA modifiers.

**FIGURE 2:**
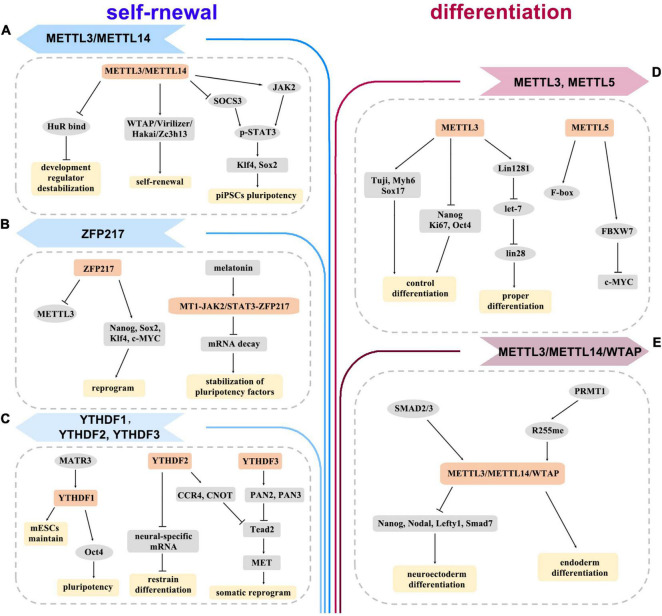
The diagram depicts the roles of m^6^A methylation in self-renewal **(A–C)** and differentiation **(D,E)** of embryonic stem cells (ESCs) and induced pluripotent stem cells (iPSCs). It proposes how writers, erasers, readers, and other regulators are involved in the regulation of various genes and pathways.

Thousands of transcripts, especially those controlling the core network during differentiation, are m^6^A modified in ESCs (Batista et al., 2014). m^6^A deposit may target pluripotency or lineage-commitment genes to regulate differentiation in different ESC state (Geula et al., 2015; Zhao and He, 2015). On one side, the absence of methyltransferase led to a dramatic differentiation defect in the naive ESCs. For instance, METTL3-depleted ESCs had enhanced self-renewal but hindered differentiation abilities (Batista et al., 2014; Geula et al., 2015; Figure 2D). In addition, METTL5 depletion led to the reduction of FBXW7 levels, thereby delaying the onset of ESC differentiation (Xing et al., 2020; Figure 2D). Moreover, Linc1281 targeted let-7/Lin28 to ensure ESC differentiation by internal m^6^A modification (Yang et al., 2018; Figure 2D). Arginine methylation of METTL14 greatly enhanced global m^6^A levels and promoted ESC endoderm differentiation (Liu et al., 2021b; Wang Z. et al., 2021; Figure 2E). On the other side, removing m^6^A triggers differentiation when ESCs are at the primed state. Loss of m^6^A methylation increased the stability of developmental genes by binding to HuR RNA to facilitate ESC differentiation (Wang et al., 2014; Figure 2A). The methyltransferase complex of ZC3H13–WTAP–Virilizer–Hakai could facilitate ESC self-renewal (Wen et al., 2018; Figure 2A). Additionally, ZFP217 modulated m^6^A deposition on Nanog, Sox2, Klf4, and c-Myc mRNAs by sequestering METTL3 (Aguilo et al., 2015; Figure 2B). Melatonin was shown to prevent m^6^A-dependent core pluripotency mRNA decay through the MT1-JAK2/STAT3-ZFP217 signal axis (Yang et al., 2020; Figure 2B). YTHDC1 was required for the maintenance of mouse ESCs (Liu et al., 2021; Figure 2C).

In the field of regeneration, whether transplanting ESCs directly or implanting organoids differentiated from ESCs, the key lies in the precise regulation of ESC proliferation and differentiation. The targets of m^6^A involve numerous genes in the network of ESC reprogramming. Understanding the mechanism of m^6^A undoubtedly helps the development of regenerative medicine.

### Induced Pluripotent Stem Cells

Induced pluripotent stem cells (iPSCs) are ESC-like cells generated by somatic cell nuclear transfer. They have similar morphology, gene expression, differentiation ability, epigenetic modification status, and function to ESCs. In-depth understanding of the mechanisms controlling self-renewal and transitions to a differentiated state is essential for iPSC in the field of regenerative medicine (Wu et al., 2019b).

However, the effects of m^6^A modification on the generation of iPSCs are inconsistent. METTL3 maintained pluripotency of porcine iPSCs by sustaining JAK2 and inhibiting SOCS3 expression, and activating STAT3/Klf4/Sox2 signal axis in an m^6^A-YTHDF1/2-dependent manner (Wu et al., 2019b; Figure 2A). MATR3 favored the pluripotency of iPSCs by regulating the transcriptional and translational efficiency of Nanog, Lin28a, and Oct4 (Pollini et al., 2021; Figure 2C). YTHDF2 was highly expressed in iPSCs and downregulated during neural differentiation. Depletion of YTHDF2 led to loss of pluripotency of iPSCs and stabilization of neural-specific gene expression (Heck et al., 2020; Liu et al., 2020; Figure 2C). SMAD2/3 facilitated cotranscriptional recruitment of METTL3/METTL14/WTAP complex onto nascent transcripts, which allowed iPSCs to timely exit from pluripotency and induced neuroectoderm differentiation (Bertero et al., 2018; Figure 2E). Thus, m^6^A signaling can induce cellular responses, which provide a basis for the clinical application of iPSCs. iPSCs avoid the ethical barriers of ESCs, which are an important advantage for clinical application. With the in-depth study of the mechanism for m^6^A regulation, iPSCs will be more widely used in regenerative medicine.

### Adult Stem Cells

#### Hematopoietic Stem/Progenitor Cells

m^6^A signal could regulate the generation of hematopoietic stem/progenitor cells (HSCs/HSPCs). In METTL3-deficient zebrafish embryos, the emergence of HSPCs was blocked (Zhang C. et al., 2017). Similar phenomena could also be observed in mouse embryonic development model. METTL3 facilitated m^6^A methylation on Notch1 to inhibit Notch activity, thereby promoting HSPC generation (Lv et al., 2018).

m^6^A signal could also regulate the development of HSCs/HSPCs. METTL3 knockdown in HSPCs promoted differentiation, while reducing proliferation (Vu et al., 2017). Conversely, another study showed that deletion of METTL3 led to an accumulation of HSCs in the bone marrow and a blockade of HSC differentiation (Lee et al., 2019). Furthermore, METTL14 knockdown substantially promoted terminal myeloid differentiation of HSPCs (Weng et al., 2018).

YTHDF2 had an important role in long-term HSC maintenance. Repression of YTHDF2 resulted in loss of lymphoid potential and HSCs expansion, as shown by the increased expression of multiple key transcription factors critical for self-renewal (Li et al., 2018; Mapperley et al., 2021). In addition, METTL3 maintained the symmetric commitment and identity of HSPCs by regulating MYC mRNA stability (Cheng et al., 2019).

#### Spermatogonial Stem Cells

Spermatogonial stem cells (SSCs) are primordial spermatogonial cells locating on the basement membrane of convoluted tubules, which can directionally differentiate into spermatocytes. METTL3 or METTL14 maintained SSC homeostasis through methylating transcripts of key regulators governing proliferation and differentiation (Lin et al., 2017). METTL3 deletion in germ cells prevented spermatogonial differentiation and meiosis (Xu et al., 2017). WTAP-mediated m^6^A modification sustained the SSC niche and governed normal spermatogenesis (Jia et al., 2020).

#### Neural Stem Cells

Neural stem cells (NSCs) exist in the nervous system and have the potential to differentiate into neurons, astrocytes, and oligodendrocytes, thus, producing a large number of brain tissue cells. m^6^A depletion by lacking METTL14 or METTL3 displayed markedly decreased proliferation in NSCs (Yoon et al., 2017; Wang et al., 2018; Chen et al., 2019). However, another study revealed that loss of METTL14 resulted in the nuclear accumulation of neural differentiation-related mRNAs, resulting in delayed neural progenitor differentiation (Edens et al., 2019).

#### Mesenchymal Stem Cells

Mesenchymal stem cells (MSCs) exist in a variety of tissues, such as the bone marrow, umbilical cord blood, placenta tissue, adipose tissue, etc. They have the potential to differentiate into mesenchymal or non-mesenchymal cells and have a unique cytokine profile. METTL3 was upregulated in bone marrow MSCs (BM-MSCs) undergoing osteogenic induction (Tian et al., 2019). It induced BM-MSC osteogenic differentiation by facilitating M1 macrophage differentiation and through the PTH/Pth1r–PI3K–AKT signaling axis (Wu Y. et al., 2018; Lei et al., 2021). ALKBH5 accelerated the degradation of PRMT6 mRNA and inhibited the osteogenic differentiation of MSCs (Li Z. et al., 2021). Another study showed that deletion of METTL3 in porcine BM-MSCs could promote the activation of the JAK1/STAT5/C/EBPβ pathway and adipogenesis in an m^6^A-YTHDF2-dependent manner (Yao et al., 2019). Furthermore, METTL3 regulated the cell cycle and mitosis by targeting PLK1, and its knockdown induced cell apoptosis and senescence in immature dental pulp MSCs (Luo et al., 2021).

#### Adipose-Derived Stem Cell

Increasing evidence show that m^6^A regulates adipose-derived stem cell (ADSC) fate decision. FTO controlled RUNX1T1 expression by regulating m^6^A levels around splice sites and thereby promoted adipogenesis (Zhao et al., 2014). Similarly, FTO depletion decreased the expression of CCNA2 and CDK2, impaired cell cycle progression, and activated JAK2-STAT3-C/EBPβ signaling, which inhibited the adipogenesis of preadipocytes (Wu R. et al., 2018; Wu et al., 2019a). In addition, ZFP217 knockdown increased METTL3 expression and the m^6^A level of CCND1, leading to the mitotic clonal expansion and adipogenesis inhibition (Liu et al., 2019).

#### Other Adult Stem Cells

Knockdown of METTL3 promoted muscle stem cell proliferation and muscle regeneration by activating Notch signaling pathway to regulate mRNA translation (Liang et al., 2021). METTL14-mediated cytoplasmic export of circGFRa1 promoted female germline stem cell self-renewal through the miR-449/GDNF signal (Li X. et al., 2021. METTL3 or YTHDF1 enhanced the translation of TEAD1 and sustained the stemness of intestinal stem cells (Jiang et al., 2021).

### Cancer Stem Cells

**FIGURE 3:**
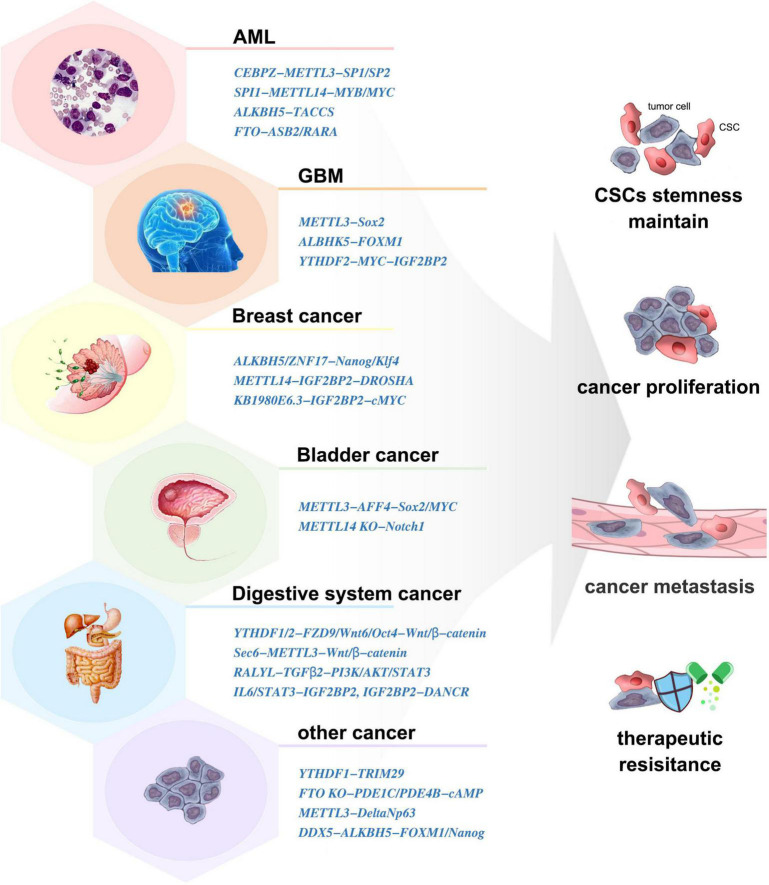
The involvement of m^6^A in various cancer stem cells (CSCs).

Cancer stem cells (CSCs) are a small group of cells with the unique property of infinite proliferation, transient amplifying, and remaining in dormancy for a long time. They express a variety of drug-resistant molecules and are not sensitive to external physical and chemical factors, which is the root of tumor proliferation, migration, and drug resistance. Dynamic m^6^A modification has been demonstrated to be involved in CSC generation and maintenance (Figure 3).

#### Acute Myeloid Leukemia

Acute myeloid leukemia (AML) is a clonal hematopoietic disorder where the differentiation capacity of HSCs/HPCs is blocked (Coombs et al., 2015). m^6^A mediates myeloid differentiation, which will provide new ideas for the pathogenesis, clinical diagnosis, and treatment of AML.

METTL3/14 was expressed more abundantly in AML cells than in healthy HSCs/HPCs, which contributed to the leukemia progression *in vivo* (Vu et al., 2017; Weng et al., 2018). Similarly, downregulation of METTL3 resulted in differentiation of leukemic cells and failure to establish leukemia in mice (Barbieri et al., 2017). Moreover, the SPI1-METTL14-MYB/MYC signaling axis was critical for the maintenance of AML and self-renewal of leukemia stem/initiation cells (LSCs/LICs) (Weng et al., 2018). ALKBH5 and FTO were also aberrantly overexpressed in AML and correlated with poor prognosis (Shen et al., 2020; Huff et al., 2021). They exerted tumor-promoting effects by posttranscriptional regulation of its critical targets such as TACC3 (Li et al., 2017; Shen et al., 2020). Inhibition of YTHDF2 specifically impaired LSCs propagation (Paris et al., 2019).

Targeting m^6^A-related molecules selectively compromised LSC proliferation and AML initiation without derailing normal hematopoiesis damage, which implied the wide application of m^6^A in the treatment of this hematological malignancy. Furthermore, targeting m^6^A-meidated control of AML cell differentiation is a promising strategy for AML therapy given the success of ATRA/arsenic trioxide (ATO)-based differentiation therapy.

#### Glioblastoma

The rapid growth and recurrence of glioblastoma (GBM) is closely related to the existence of glioblastoma stem cells (GSCs). m^6^A modification peaks were enriched at metabolic pathway-related transcripts in GSCs compared with neural progenitor cells (Li et al., 2019), which implied a crucial role of m^6^A methylation for GSCs.

The elevated expression of METTL3 was indispensable in the execution of oncogenic pathways and clinical aggressiveness (Li et al., 2019; Visvanathan et al., 2019). METTL3-silenced GSCs showed significant rescue in neurosphere formation and inhibited tumor growth *in vivo* (Visvanathan et al., 2018). Mechanically, METTL3 sustained its oncogenic role by enhancing the stability and expression of Sox2 (Visvanathan et al., 2018), altering A-to-I and C-to-U RNA-editing events (Visvanathan et al., 2019), and modulating mRNA decay of splicing factors and alternative splicing isoform switches (Li et al., 2019). However, some studies showed that knockdown of METTL3/14 dramatically increased GSC growth and self-renewal (Cui et al., 2017). The abnormally high expression of ALKBH5 has been detected in GSCs. ALKBH5 demethylates FOXM1 nascent transcripts, leading to GSC tumorigenesis (Zhang S. et al., 2017). In contrast to NSCs, GSCs displayed preferential expression of YTHDF2, laying the foundation for the YTHDF2–MYC–IGF2BP3 axis as a novel therapeutic target in GBM (Dixit et al., 2021). The FTO inhibitors could prevent neurosphere formation in GSCs without inhibiting the growth of healthy neural stem cells (Huff et al., 2021). m^6^A induced neuroshere formation and γ-irradiation resistance and promoted GBM growth, which makes it an important target for GBM diagnosis and treatment. Clarifying the expression and mechanism of m^6^A-related molecules in GCS formation is helpful in the finding and design of small-molecule inhibitors. Verifying the roles of these inhibitors in clinical application is an important research direction in the future.

#### Breast Cancer

Breast cancer stem cells (BCSCs) is specified by the expression of core pluripotency factors, including Oct4, Sox2, Klf4, and Nanog. ALKBH5 was stimulated in breast cancer cells when exposed to hypoxia, which induced the BCSC phenotype and increased the number of BCSCs by demethylating Nanog (Zhang et al., 2016a). Exposure of BCSCs to hypoxia induced ZNF217-dependent inhibition of m^6^A methylation on Nanog and Klf4 (Zhang et al., 2016b). LncRNA KB-1980E6.3 recruited IGF2BP2 to enhance c-MYC stability to maintain the stemness of BCSCs (Zhu et al., 2021). The AURKA–METTL14–IGF2BP2 complex promoted and stabilized DROSHA methylation to maintain BCSC stemness (Peng et al., 2021). Thus, m^6^A played an irreplaceable role in maintaining the phenotype and quantity of BCSCs. Targeting m^6^A-related molecules or mechanisms is an effective way to clear BCSCs and inhibit breast cancer.

#### Bladder Cancer

Global RNA m^6^A abundance and the expression of METTL3 was higher in CSCs than those in non-CSCs of bladder cancer cells. METTL3 regulated the m^6^A modification and expression of AFF4, which bound to the promoter regions and sustained the transcription of Sox2 and MYC, to promote self-renewal of bladder cancer CSCs (Gao et al., 2020). In contrast, METTL14 was lowly expressed in bladder tumor-initiating cells (TICs). METTL14 inhibited the proliferation, self-renewal, metastasis, and tumor-initiating capacity of TICs by participating in the RNA stability of Notch1 mRNA (Gu et al., 2019).

#### Digestive System Tumor

Recent studies have shown that m^6^A plays important roles in the regulation of digestive system CSCs. In colorectal cancer, YTHDF1 promoted the translation of m^6^A-modified FZD9 and Wnt6, leading to aberrant activation of Wnt/β-catenin signaling and ultimately increasing the number of CSCs (Bai et al., 2019). Low FTO expression elevated m^6^A levels, resulting in enhanced CSC tumorigenicity and chemoresistance (Relier et al., 2021). METTL3 upregulated Sec62 to promote the stemness and chemoresistance of CSCs by enhancing Wnt/β-catenin signaling (Liu et al., 2021a). In hepatocellular carcinoma, YTHDF2 promoted cancer metastasis and the emergence of CSCs by modulating the m^6^A methylation of Oct4 (Zhang et al., 2020). RALYL enhanced CSC stemness by sustaining TGF-β2 mRNA stability (Wang X. et al., 2021). In pancreatic cancer, IGF2BP2 and DANCR worked together to promote CSC properties (Hu et al., 2020). In cholangiocarcinoma, IL-6/STAT3 triggered inflammatory response to facilitate CSC stemness through regulating m^6^A writers (Ye et al., 2021). These results showed that m^6^A-related molecules might provide potential therapeutic targets in these tumors. However, further research is needed to explore the roles and mechanisms of m^6^A in CSCs of more digestive system tumors.

#### Other Tumors

Recruitment of YTHDF1 to m^6^A-modified TRIM29 was involved in promoting TRIM29 translation and enhancing the CSC characteristics in cisplatin-resistant ovarian cancer cells (Hao et al., 2021). Conversely, FTO could augment cAMP signaling and suppressed CSC features of ovarian cancer (Huang et al., 2020). m^6^A methylation in multidrug-resistant osteosarcoma cells was found to correlate with the emergence and maintenance of osteosarcoma CSCs (Wang et al., 2019). The deletion of METTL3 impaired cutaneous squamous cell carcinoma CSC properties, including colony-forming ability *in vitro* and tumorigenicity *in vivo* (Zhou et al., 2019). In oral squamous cell carcinoma, ALKBH5 regulated by DDX3 led to decreased m^6^A methylation in FOXM1 and Nanog, which contributes to increased CSC population (Shriwas et al., 2020).

## Prospect of Clinical Application and Future Research

Stem cells perform complex biological processes through the dynamic changes of pluripotency or pedigree factors. Their gene expression programs need not only sufficient stability to maintain self-renewal and pluripotency over multiple cell generations but also flexibility to change rapidly in response to differentiation cues (Batista et al., 2014). The reversible change in m^6^A marks turns over transcript expression in a timely fashion, which becomes an effective means to regulate stem cell fate (Batista et al., 2014). m^6^A maintain the balance between pluripotency and lineage priming factors, thus, ensuring orderly differentiation of stem cells (Batista et al., 2014; Geula et al., 2015; Zhao and He, 2015). m^6^A may intersect with other preexisting pathways by altering downstream gene expression to regulate the stem cell differentiation network (Geula et al., 2015; Zhao and He, 2015). m^6^A could also target non-coding RNAs to regulate stem cell fate through existing mechanisms. The detailed classification of stem cells is based on different species or tissue sources, states, external environment, differentiation stages, and so on. These factors may lead to different outcomes of m^6^A modification. Therefore, further studies are needed to explore the specific molecular mechanism in different types of stem cells (Liang et al., 2020).

As a switch regulator of stem cell self-renewal and differentiation, m^6^A has great potential in the clinical application of stem cells. (1) ESCs can be used as seed cells for cell therapy and organ replacement therapy. It is a difficult problem for stem cells to overcome aging and maintain long-term self-renewal. METTL3 ablation and m^6^A loss increase the stability of methylated pluripotent mRNA transcripts and contribute to the pluripotent phenotype of ESCs (Geula et al., 2015). It is possible for damaged tissues or organs to recover completely by regulating the artificial expansion *in vitro* and directional differentiation *in vivo* of stem cells through m^6^A modification. (2) The establishment of human embryonic stem cell line provides an important tool to explore the influencing factors and regulatory mechanisms in the process of body development. It is expected to reveal the molecular mechanism of embryonic development by comparing the differences of m^6^A status and gene expression between ESCs and differentiated cells in different time and space. (3) Stem cells are ideal carriers for the gene therapy of diseases and have broad application prospects in the fields of severe immune deficiency, genetic diseases, malignant tumors, AIDS, and so on. The progress of stem cell isolation and purification technology, the improvement of gene transfection efficiency, the continuous improvement of gene transfer vector, the expansion and directional differentiation of transgenic cells, the discovery of new target genes, the stable expression and regulation of target genes after transfection, and other fields have attracted the attention of scientists. m^6^A cannot only regulate the phenotype of transgenic stem cells but also regulates the expression of target genes through posttranscriptional modification. (4) The involvement of m^6^A in CSCs may be used for predicting cancer risk, achieving early diagnosis, tracking the prognosis of tumor fate, and ultimately, providing novel therapeutic approaches. Evidence has highlighted the potential of both the altered m^6^A levels and the score model with different methylase as promising biomarkers in various cancers, such as lung cancer, prostatic cancer, bladder cancer, AML, and so on (Ma and Ji, 2020). Currently, inhibitors of FTO and ALKBH5 are being used as candidates for anticancer drug development, especially to inhibit the growth of CSCs by manipulating their m^6^A modification levels. Although these inhibitors have not been tested in clinical trials, they will provide more possibilities for the treatment of cancer (Xu et al., 2020).

So far, the information on m^6^A in stem cell fate regulation is still lacking. A wider scope of research should be considered: (1) New m^6^A writers, erasers, readers, and associated effectors need to be identified. Discovery of more components could help understand the regulatory network of m^6^A. (2) Once these new members are identified, their cell location, biological structure, physical and chemical properties, physiological functions, and other characteristics need to be further studied. (3) Determining the additional factors that coordinate m^6^A deposition or removal, and understanding how these factors are regulated and how the specificity of the methylated sites is achieved. (4) Verification of existing m^6^A components or functions in more biological events. (5) More precise techniques are needed to assist in the study of the distribution and target selection of m^6^A. (6) Further exploring diagnostic index and therapeutic targets involved in m^6^A machinery complexes might be very promising for some stubborn diseases, such as cancers or neurological diseases. These extensive studies may unveil more exact mechanisms of m^6^A in multiple biological processes.

## Conclusion

Current studies on posttranscriptional modifications have revealed another layer of complexity in the regulation of cellular processes. In this review, we have summarized current knowledge on m^6^A and described their roles in stem cell self-renewal and differentiation. We put forward the potential of m^6^A mechanism in the clinical application of stem cells in future studies. Despite the progress made in recent years, more studies are still needed to provide clear information for the functional roles of m^6^A modification and the underlying mechanisms in stem cell fate decision.

## Author Contributions

RJ wrote the manuscript and designed the figures. XZ revised the manuscript. Both authors read and approved the final manuscript.

## Conflict of Interest

The authors declare that the research was conducted in the absence of any commercial or financial relationships that could be construed as a potential conflict of interest.

## Publisher’s Note

All claims expressed in this article are solely those of the authors and do not necessarily represent those of their affiliated organizations, or those of the publisher, the editors and the reviewers. Any product that may be evaluated in this article, or claim that may be made by its manufacturer, is not guaranteed or endorsed by the publisher.

## Abbreviations

METTL3/5/14/16: methyltransferase-like 3/5/14/16
WTAP: WT1-associated protein
RBM15/15B: RNA-binding motif protein 15/15B
ZC3H13: zinc finger CCCH-type-containing 13
FTO: alpha-ketoglutarate-dependent dioxygenase
ALKBH5: AlkB homolog 5, RNA demethylase
YTHDF1/2/3: YTH N6-methyladenosine RNA-binding protein 1/2/3
YTHDC1/2: YTH domain-containing 1/2
IGF2BP1/2/3: insulin-like growth factor 2 mRNA-binding protein 1/2/3
HNRNPA2B1/C/G: heterogeneous nuclear ribonucleoprotein A2B1/C/G
LC-MS/MS: liquid chromatography coupled to tandem mass spectrometry
MeRIP-seq: methylated RNA immunoprecipitation sequencing
PA-m^6^A-CLIP: photo-crosslinking-assisted m6A sequencing strategy
miCLIP: m6A individual-nucleotide-resolution crosslinking and immunoprecipitation
m6A-LAIC-SEQ: m^6^A-level and isoform-characterization sequencing
SCARLET: site-specific cleavage and radioactive-labeling followed by ligation-assisted extraction and thin-layer chromatography
SELECT: single-base elongation and ligation-based qPCR amplification method
ESC: embryonic stem cell
Nanog: Nanog homeobox
Klf4: Kruppel-like factor 4
MYC: MYC proto-oncogene, BHLH transcription factor
Lin28: Lin-28 homolog
Med1: mediator complex subunit 1
Jarid2: jumonji and AT-rich interaction domain-containing 2
Eed: embryonic ectoderm development
FBXW7: F-box and WD repeat domain-containing 7
ZFP217/ZNF217: zinc finger protein 217
Sox2: SRY-box transcription factor 2
JAK2: Janus kinase 2
STAT3: signal transducer and activator of transcription 3
iPSC: induced pluripotent stem cell
PAN2/3: poly(A) specific ribonuclease subunit PAN2/3
Tead2: TEA domain transcription factor 2
TGFβ1: transforming growth factor beta 1
SOCS3: suppressor of cytokine signaling 3
SMAD2/3: SMAD family member 2/3
HSC/HSPC: Hematopoietic stem/progenitor cell
HE: hemogenic endothelial
DA: dorsal aorta
EHT: endothelial-to-hematopoietic transition
SSC: spermatogonial stem cell
NSC: neural stem cell
FMRP: fragile X mental retardation protein
MSC: mesenchymal stem cell
PTH/Pth1r: parathyroid hormone/parathyroid hormone receptor-1
PI3K: phosphatidylinositol-4,5-bisphosphate 3-kinase
AKT: AKT serine/threonine kinase 1
PLK1: polo-like kinase 1
EBPβ: CCAAT enhancer-binding protein beta
ADSC: adipose-derived stem cell
RUNX1T1: RUNX1 partner transcriptional corepressor 1
CCNA2: cyclin A2
CDK2: cyclin-dependent kinase 2
CCND1: cyclin D1
CSC: cancer stem cell
AML: acute myeloid leukemia
CEBPZ: CCAAT enhancer-binding protein zeta
ATRA/ATO: all-trans-retinoic acid/arsenic trioxide
LSC/LIC: leukemia stem/initiation cell
TACC3: transforming acidic coiled-coil-containing protein 3
ASB2: ankyrin repeat and SOCS box containing 2
RARA: retinoic acid receptor alpha
GBM: glioblastoma
GSC: glioblastoma stem cell
HuR: human antigen R
ADAR: adenosine deaminase RNA specific
APOBEC3A: apolipoprotein B MRNA-editing enzyme catalytic subunit 3A
FOXM1: forkhead box M1
FOXM1-AS: a long non-coding RNA antisense to FOXM1
Oct4: octamer-binding transcription factor 4
BCSC: breast cancer stem cell
HIF: hypoxia inducible factor
DROSHA: Drosha ribonuclease III
STC1: stanniocalcin 1
AURKA: Aurora kinase A
AFF4: AF4/FMR2 family member 4
FZD9: frizzled class receptor 9
PCIF1/CAPAM: phosphorylated CTD-interacting factor 1
TRIM29: tripartite motif containing 29
PDE1C/4B: phosphodiesterase 1C/4B
cAMP: cyclic adenosine monophosphate
DDX3: DEAD-box helicase 3.
